# LATER-ADULTHOOD TRAUMA REENGAGEMENT IN VIETNAM VETERANS WITH PTSD: FINDINGS FROM A PROGRAM EVALUATION PROJECT

**DOI:** 10.1093/geroni/igz038.2455

**Published:** 2019-11-08

**Authors:** Kelly A O Malley, Patricia M Bamonti, Kirsten Graham, Ronald Smith, Jacqueline Gurevitch, Anica Pless Kaiser

**Affiliations:** 1 New England GRECC, Boston VA Healthcare System, Boston, Massachusetts, United States; 2 VA Boston Healthcare System, Boston, Massachusetts, United States; 3 National Center for PTSD, Behavioral Sciences Division VA Boston Healthcare System, Boston, Massachusetts, United States

## Abstract

As Veterans age, they may experience an emergence or exacerbation of stress symptomatology. Later-Adulthood Trauma Reengagement (LATR) is an intervention that provides psychoeducation on the LATR process, teaches mindfulness and coping, facilitates trauma re-integration, and fosters meaning-making in late life. This program evaluation project evaluated the LATR group offered in the Geriatric Mental Health Clinic at VA Boston. Twenty-one Vietnam Veterans with PTSD symptomatology were referred to the group between 2017-2018. Fifteen completed the 10-week group, and 12 completed pre- and post-intervention assessments and offered feedback. At the conclusion of the group, Veterans reported a decrease in depressive and PTSD symptoms. Of the 4 Veterans who endorsed suicidal ideation at the start of the group, 3 reported a decrease post-group. Veterans endorsed slightly more positive appraisals of their military service. Veterans reported that the group offered support, connection, and a sense of belonging with other Veterans, helped increase coping, and increased insight into thoughts, feelings, and behaviors. A suggested improvement to the group was to offer more sessions. Eleven Veterans sought continued mental health services following the group; three newly sought trauma-focused interventions. Four Veterans reported that their trauma-related symptoms were resolved and declined referral. Veterans found the LATR group helpful as evidenced by reduction in symptoms and responses to open-ended questions. A small number sought additional trauma-focused treatment; others reported that their symptoms were resolved and did not require additional treatment. Findings support the continued offering of the LATR group within this clinical setting.

